# Optimization of 3D Printing Nozzle Structure and the Influence of Process Parameters on the Forming Performance of Underwater Concrete

**DOI:** 10.3390/ma18071431

**Published:** 2025-03-24

**Authors:** Kun Yang, Jingbo Yuan, Yibo Wang, Fan Yang, Changzai Ren

**Affiliations:** 1 School of Mechanical Engineering and Automation, Liaoning University of Technology, Jinzhou 121000, China; jxyangkun@lnut.edu.cn (K.Y.); lntu163@163.com (Y.W.); yf18829030686@163.com (F.Y.); rcz@qlu.edu.cn (C.R.); 2 School of Energy and Power Engineering, Qilu University of Technology, Jinan 250316, China

**Keywords:** forming quality, mechanical property, nozzle structure optimization, process parameters, underwater 3D printing concrete

## Abstract

Underwater concrete 3D printing (3DPC) technology, as a pioneering construction process, has demonstrated significant potential in various fields, such as marine engineering, underwater restoration projects, and ecological construction. However, the complexity and variability of the underwater environment pose stricter quality standards for the printed structures. To address this, this study employed a self-developed framed concrete 3D printer and utilized response surface methodology to optimize the structural dimensions of the printing nozzle. Through in-depth analysis of the internal flow field of printing nozzles with various size combinations using ANSYS Fluent 2022R1 software, an optimal parameter configuration was determined, including a nozzle diameter (*D*) of 55 mm, an inclination angle (*θ*) of 20°, and a length (*L*) of 34 mm, ensuring uniform extrusion of the concrete material. Furthermore, this study applied an orthogonal experimental design to systematically investigate the combined effects of screw speed, printing speed, and nozzle height on the print quality and mechanical properties (compressive strength and flexural strength) of underwater concrete 3D printing. The experimental results, presented based on direct observation and analysis, identified the optimal combination of process parameters: a printing speed of 16 mm/s, a nozzle height of 10 mm, and a screw speed of 50 r/min. This combination ensures efficient printing while maintaining the mechanical properties of the printed samples. This study not only provides solid scientific and practical guidance for optimizing the nozzle structure and process parameters of underwater concrete 3D printing technology but also offers innovative solutions to underwater construction challenges in the field of marine resource development and utilization.

## 1. Introduction

With the gradual deepening of human exploitation and utilization of water resources, underwater construction is becoming more and more common [1,2,3]. Three-dimensional printing concrete technology is a forming method that selectively superimposes concrete materials using a print nozzle. Among them, this technology provides a new idea for the development of a green construction industry with the characteristics of digital manufacturing, stacking manufacturing, direct manufacturing, and rapid manufacturing [4]. Three-dimensional printing technology has now broken through the restrictions on land and is expected to be applied to underwater engineering. Three-dimensional printing architecture under water is completely different from traditional underwater construction methods, which also puts higher requirements on concrete materials [5,6,7]. It is necessary to have excellent constructability, underwater dispersion resistance, high mechanics and durability, as well as appropriate operability and rapid hardening ability to ensure the stability, safety, and environmental protection of the underwater printing structure.

At present, there are few reports on 3D printing of underwater concrete at home and abroad. Brahim Mazhoud et al. [2] evaluated the underwater 3D printing of cement-based materials and found that increasing the printing speed will reduce the compressive strength but increase the elastic modulus (below the critical speed). Sun Xiaoyan et al. [8] optimized the mix ratio of underwater 3D-printed concrete and found that increases in the water-binder ratio, ore-powder ratio and sand-binder ratio reduced the compressive strength. Additionally, the optimal flocculant content was 2%. They analyzed the influence of printing process parameters on interlayer bonding, established a model for the change in bonding strength with printing time interval, and studied the influence of nozzle parameters on interlayer bonding. A degradation model of interlayer bond strength with printing speed was proposed. Wang et al. [9] discussed the effects of printing environment and construction methods on the mechanical properties of underwater 3D-printed concrete. The results showed that compared with the cast samples in the air, the printing method and underwater environmental factors caused the compressive strength of U3DPC to decrease by 20% and 15.1%, respectively. Seo et al. [10] used 3D printing technology to print concrete containing coarse aggregate and PP fiber and found that the samples printed in air were denser and had higher compressive strength than the poured samples. Yang et al. [11] studied the effects of various anti-dispersants and polymers on the properties of 3DPC. Rahul et al. [12] pointed out that the nozzle size, pumping flow rate, extrusion speed, and nozzle movement speed have significant effects on the shape of the printed mortar strip. It is suggested to use a rectangular nozzle and match the extrusion speed and nozzle movement speed to keep the shape stable. Tay et al. [13] used a 30 mm × 15 mm rectangular nozzle and found that the flow rate and moving speed affected the cross-section shape. In addition, these researchers introduced the SR parameter (the ratio of the actual cross-section area to the nozzle area) and found that the printing effect was the best when the SR was close to 1. Paul et al. [14] found in their research that, compared to rectangular nozzles, circular nozzles can adapt to printing processes at different rotational angles. However, the gaps in the 3D-printed concrete using circular nozzles are significantly larger, and the mechanical strength is poorer. As the nozzle size increases, the mechanical strength of the 3D-printed concrete also increases. Panda et al. [15] demonstrated in their study that the tensile strength of the interface of printed mortar decreases with increasing printing speed and nozzle height, with printing speed having a greater impact. When the printing speed increases from 70 mm/s to 110 mm/s, the tensile strength of the interface decreases by 14%.

To sum up, the current systematic research on the structural optimization of underwater 3D printing concrete printing nozzles and the effect of process parameters on forming quality and performance has not been reported, which needs to be further explored and expanded. In concrete 3D printing technology, the print nozzle should not only meet the needs of the smooth extrusion of the homogeneous slurry, but also ensure that the printed components do not collapse and have good interlayer bonding strength. Therefore, the structure optimization of the printing nozzle is also one of the important tasks in the development of the equipment. In this paper, a self-developed frame concrete 3D printer was used as a platform to study the influence of the main parameters of the printing nozzle on the exit speed using numerical simulation, and the response surface method was used to optimize the structure size, so as to improve the printing accuracy and surface quality. At the same time, an orthogonal experiment method was used to study the influence of screw speed, printing speed, nozzle height, and other process parameters on the forming quality and mechanical properties of underwater concrete 3D printing. The coupling between the process parameters was analyzed, and the best process parameters suitable for underwater 3D printing concrete were determined, providing a certain reference for the popularization and application of underwater concrete printing.

## 2. Underwater 3DPC Experimental Materials and Test Methods

### 2.1. Raw Materials and Mix Ratio

The quality of the underwater 3DPC foundation used is shown in Table 1 [8]. The admixture content is measured by the mass percentage of the gelling material. The water-binder ratio (mass ratio) is 0.28, and the sand-binder ratio (mass ratio) is 0.25.

The cementitious materials used consist of sulfoaluminate cement, ultrafine fly ash, and mineral powder. The sulfoaluminate cement (with performance parameters detailed in Table 2) is sourced from Zhucheng Jiuqi Building Materials Co., Ltd. (Zhucheng, China), in Shandong Province, while the ultrafine fly ash and mineral powder are obtained from Gongyi Water Purification Material Co., Ltd. (Gongyi, China). The aggregate employed is quartz sand with a mesh size of 40, which enhances the constructability of the 3D-printed concrete. As an anti-dispersant, hydroxypropyl methylcellulose (HPMC) with a viscosity of 200,000 cPs is used to improve the anti-dispersion and plasticity of the cement, thereby strengthening the interlayer bonding strength. A water-reducing agent (WR) is adopted to adjust the fluidity, and lithium carbonate (Li_2_CO_3_) serves as a retarder to control the setting time of the cement, ensuring a suitable operable window for 3D printing.

### 2.2. Performance Test Method

#### 2.2.1. Rheology Test

Stir the mixed cement-based material at 60 r/min for 90 s. Then, after standing for 15 s, stir the mixture at 120 r/min for 90 s. Rheological tests were carried out with a LBY-III rheometer. The test time was 700 s, and the logarithm of the shear rate increased from 1 s^−1^ to 700 s^−1^ during 0–700 s. The test conditions were 20 ± 2 °C room temperature and 65 ± 10% relative humidity (RH).

#### 2.2.2. Constructability Test

**Figure 1:**
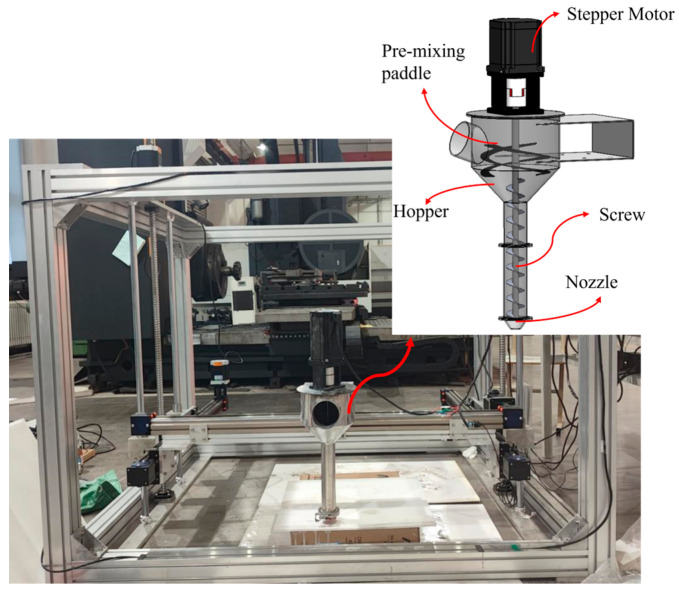
Frame concrete 3D printer.

**Figure 2:**
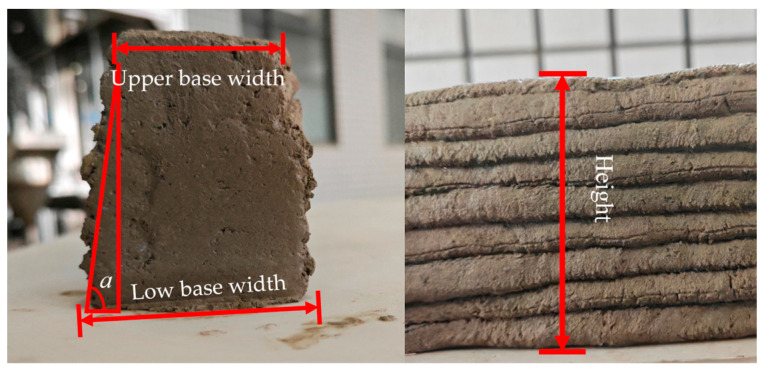
Construction performance test.

Figure 1 displays our self-designed framed concrete 3D printer, featuring a screw extrusion mechanism as its core component. This mechanism comprises a pre-mixing paddle, material hopper, screw, and nozzle. During operation, mixed concrete is poured into the material hopper, where it is maintained in a fluid state using the pre-mixing paddle before being pushed by the screw to the nozzle for printing. The model dimensions designed for the constructability experiment are 160 mm in length, 40 mm in width, and 80 mm in height, with each layer being 8 mm thick for a total of 10 layers. The printing speed remains constant throughout the process, and measurements are taken after printing to calculate the tangent of the inclination angle α and the cross-sectional area ratio. As shown in Figure 2, good constructability is characterized by a relatively high height, upper and lower base widths close to the designed width, a large inclination angle α, and a cross-sectional area ratio approaching 1. These parameters serve as criteria to evaluate the quality of constructability. Due to significant variations in the printable open time of materials, in this study, the constructability test was conducted on 3D-printed cement-based materials after 15 min of water addition.

#### 2.2.3. Anti-Dispersion Performance Test

**Figure 3:**
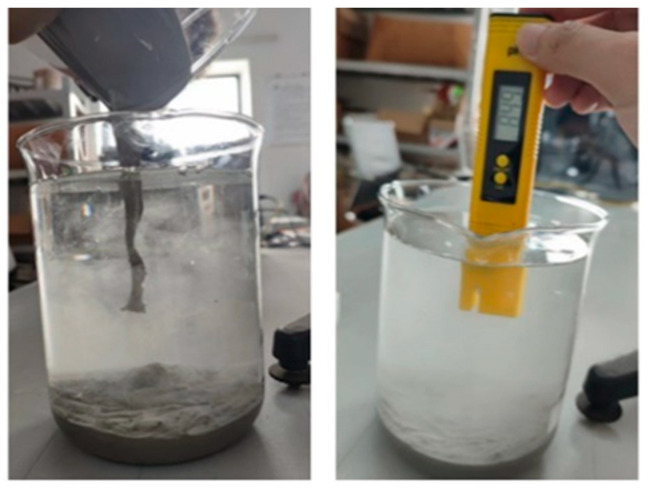
pH measurement.

The dispersion resistance of underwater 3DPC can be characterized by testing the mass loss rate of samples in water and the change of pH value in the surrounding waters [11]. pH value measurements refer to existing methods. As shown in Figure 3, 500 g mortar was slowly dropped from the water surface. After standing for 3 min, the pH value of the solution was recorded after the mortar was added to the water for 5, 10, 20, and 30 min [11]. The pH value was measured using a Milor’s pH test pen with an accuracy of 0.01.

**Figure 4:**
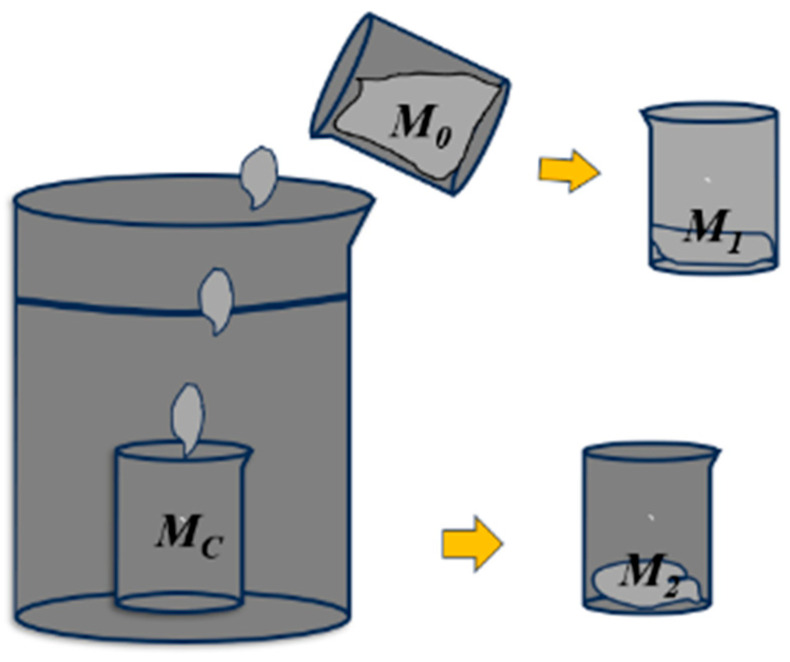
Principle diagram of mass loss rate measurement.

The test method of mass loss rate is shown in Figure 4. A beaker (mass mark *Mc*, accurate to 0.1 g) is placed in a container with an inner diameter of 180 mm and a height of 300 mm, and the water level in the container is 200 mm higher than the port on the beaker. The mixed mortar (total mass *M*_0_ of mortar and beaker, accurate to 0.1 g) is slowly and freely dropped from the water surface, so that all of it falls into the beaker placed in the container. The total mass of the residual mortar and beaker is weighed (note *M*_1_, accurate to 0.1 g), and after standing for 5 min, the beaker in the container is slowly lifted from the water to drain the water on the mortar. Wipe off the clear water on the surface of the beaker and weigh (the mass is recorded as *M*_2_, accurate to 0.1 g). Repeat the above operation three times, and take the average. The measurement is accurate to 0.01%. The formula for calculating the mass loss rate *M* is expressed as follows:

(1) $$M=\frac{M_{0}-M_{1}-M_{2}+M_{c}}{M_{0}-M_{1}}$$

#### 2.2.4. Underwater Printing Experiment

**Figure 5:**
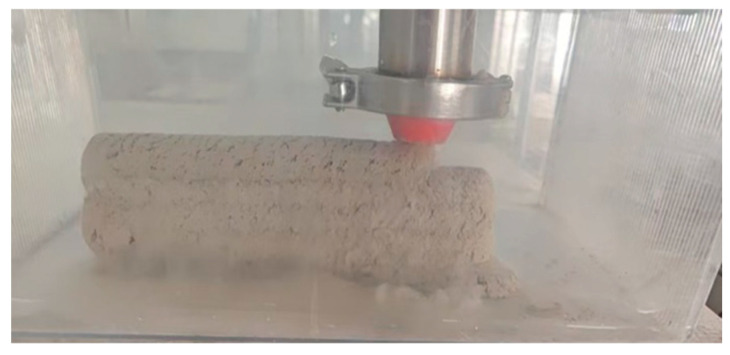
Underwater printing.

As shown in Figure 5, a transparent water tank with dimensions of 50 cm in length, 45 cm in width, and 40 cm in height is filled with water, with the water surface positioned 15 cm above the tank bottom. To more prominently demonstrate the impact of different process parameters on the quality of underwater 3D-printed concrete, a circular nozzle with an outlet diameter of 15 mm is installed here. A rectangular print model with dimensions of 220 mm in length, 45 mm in width, and 50 mm in height is designed. After setting the process parameters for each group, the printing device is activated. The entire printing process is monitored using photography. Upon completion of printing, the group with the best printing results is selected for a 7-day underwater curing process.

#### 2.2.5. Mechanics Performance Test

In this paper, a CMT-5305 electronic universal testing machine manufactured by MTS Industrial Systems (China) Co., Ltd. (Shanghai, China), was used to measure the compressive strength and flexural strength of the specimens, and the experiment was conducted in accordance with GB/T 17671-2021 [16] specification. Select well-printed specimens for compressive and flexural strength tests. The displacement control loading method is adopted, and the loading speed is 2 mm/min. The distance between the two fulcrum centers of the bending test support platform is 120 mm. The production method of the sample is as follows: put it in water, remove it when it is not deformed, pre-cut it with a cutting machine, put it in water for 7 days to remove it, and polish the surface of the sample with an angle grinder. Finally, the sample is printed with a cube of 40 mm width and height and a prism of 40 mm × 40 mm × 160 mm. The compressive strength and flexural strength of the materials were measured separately.

## 3. Print Nozzle Structure Optimization

### 3.1. Nozzle Structure Design

**Figure 6:**
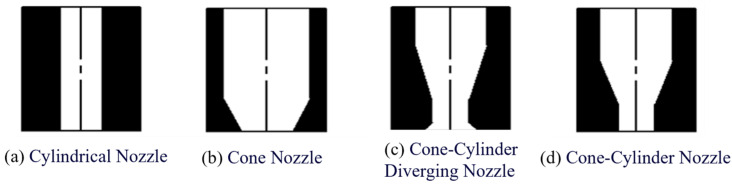
Nozzle structures.

The nozzle serves as the final outlet for the printing material, and its design has a significant impact on print accuracy. Based on their shapes, print heads can be classified into needle-type nozzles, fan-shaped nozzles, and irregular-shaped nozzles [17]. Taking into account the characteristics of various nozzle shapes, the needle-type structure is initially chosen. Within the needle-type nozzle classification, there are further subdivisions, including cylindrical nozzles, cone nozzle, cone-cylinder diverging nozzles, and cone-cylinder nozzles, as illustrated in Figure 6.

Research has found that under the same inlet and outlet conditions, both conical nozzles and cone-to-cylinder (or cone-cylindrical, if this is the term used in your context) nozzles exhibit relatively uniform outlet velocities, with relatively low turbulence intensity at the outlet and along the wall surface [18]. However, considering the convenience of subsequent processing, this paper selects the conical nozzle.

### 3.2. Nozzle Simulation Test Design

**Figure 7:**
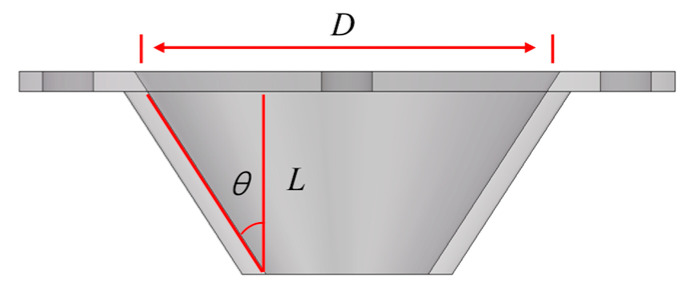
Nozzle parameters.

This paper adopts the principles of the central composite (Box–Behnken) experimental design, as shown in Figure 7. Three factors that affect the stability of the cross-sectional velocity at the outlet of the printing nozzle are selected: the inlet diameter *D* of the nozzle, the nozzle inclination angle *θ*, and the outlet length *L.* The range of values for each parameter is given in Table 3. A three-factor, three-level experimental design and analysis are conducted, with the results of the experimental design presented in Table 4.

As shown in Figure 3, the structural characteristics of the conical nozzle are mainly determined by parameters such as the inlet diameter *D*, the nozzle inclination angle *θ*, and the outlet length *L*, and the specific values of each parameter are obtained from the simulation results during the operation of the nozzle.

### 3.3. Simulation Analysis

#### 3.3.1. Model and Mesh

**Figure 8:**
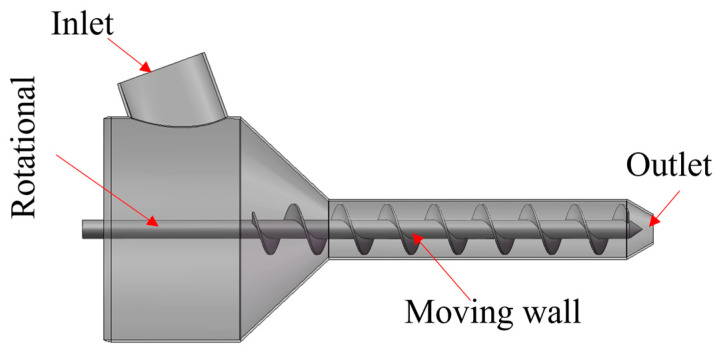
3D simulation model.

**Figure 9:**
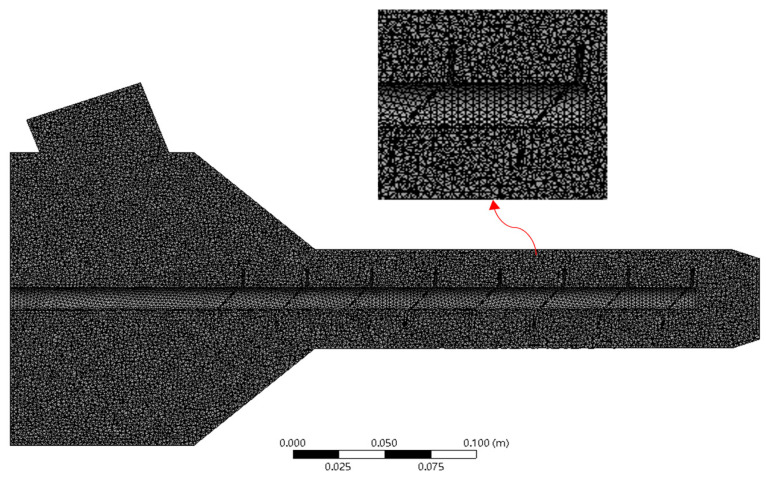
Mesh of the simulation model.

This paper utilizes the computational fluid dynamics analysis software ANSYS Fluent 2022 R1 as the platform and selects concrete as the research material. Due to the complexity of the flow, simplifications and assumptions have been made in the model. The concrete material is assumed to be a generalized non-Newtonian, incompressible fluid, and the energy equation is neglected. Gravitational work is preserved, and the output is isothermal. SolidWorks is used to create an overall model of the fluid domain, as shown in Figure 8. Tetrahedral meshes are employed to locally refine the mesh, as illustrated in Figure 9. Considering computational efficiency and time, a mesh independence validation is conducted, resulting in a mesh count of 2.2 million.

#### 3.3.2. Rheological Parameters

**Figure 10:**
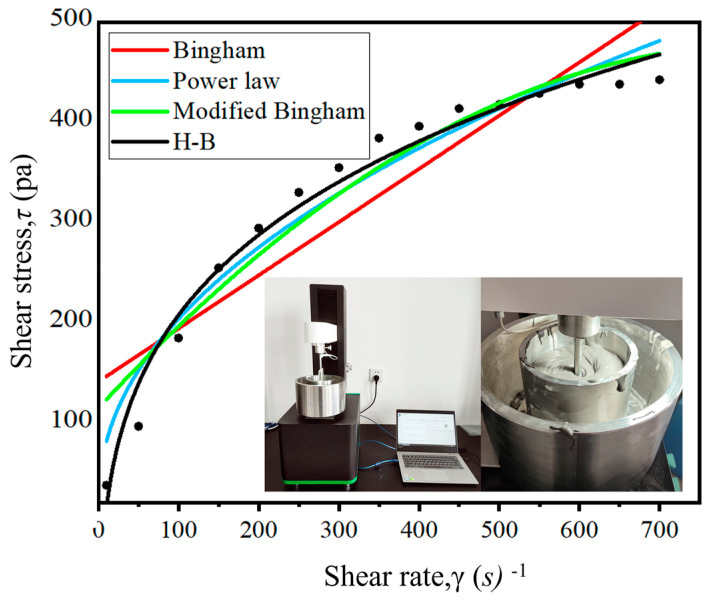
Data model fitting.

The rheological equation fitted using least squares nonlinear multivariate regression is presented in Table 5. Based on the parameters measured using the rheometer, the corresponding curves are shown in Figure 10. The correlation coefficients (R^2^) of the four equations are all greater than 0.95, except for the Bingham model. However, the most significant model is the Herschel–Bulkley (H–B) model. Considering the convenience of finite element analysis, the modified Bingham model is selected to represent the rheological characteristics of underwater 3D-printed concrete (3DPC). The error is within the confidence interval, and the rheological equation is given as follows: *τ* = 9.91 + 0.09*γ* − 5.41*γ*^2^.

#### 3.3.3. Boundary Conditions

Set the density of underwater 3D-printed concrete (3DPC) to *ρ* = 2400 kg/m^3^, and add a gravitational acceleration of 9.8 m/s^2^ in the *Z*-axis direction. Assume that the screw channel is filled with fluid, and the screw rotates at a speed of 40 revolutions per minute (r/min). Set the outer surface of the screw as a moving wall (Moving wall) with a motion form of rotation (Rotational). The remaining walls are set as stationary and no-slip walls. The inlet velocity is converted into a mass flow rate based on the screw conveyance principle, and the inlet mass flow rate q is calculated to be 0.06 kg/s using Equation (2).

(2) $$q=\frac{\pi \left(R^{2}-r^{2}\right)\cdot n\cdot S\cdot \rho }{60}$$

In this context, R represents the major diameter of the screw, measured in meters (m); r represents the minor diameter of the screw, also in meters (m); n is the rotational speed of the screw, given in revolutions per minute (r/min); S is the pitch of the screw, measured in meters (m); and ρ is the density of the concrete, measured in kilograms per cubic meter (kg/m^3^). The outlet is set as a pressure outlet with a default pressure of 0 Pa. After setting up the boundary conditions, the SIMPLE (Semi-Implicit Method for Pressure-Linked Equations) solution method is used. Initialization is performed, and the calculation is submitted.

#### 3.3.4. Simulation Result Analysis

**Figure 11:**
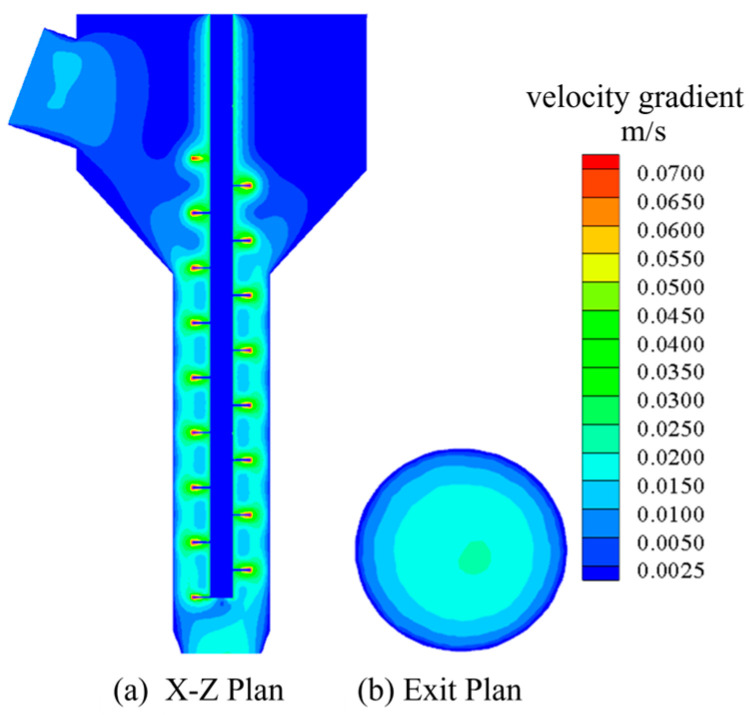
Simulation result graph.

Figure 11 presents the simulation results of Group 1. Specifically, Figure 11a presents the velocity cloud image perpendicular to the plane at the exit section, and Figure 11b presents the velocity cloud image at the exit section. It can be seen in Figure 11 that the flow velocity of concrete slurry from inlet to outlet gradually increases first and then decreases gradually. In the case of rotation of the screw, the velocity of the slurry near the screw is larger because the slurry has a certain viscosity. When the screw is agitated, the slurry near the screw will experience shear, and the movement will be carried out under the action of gravity and shear force. Meanwhile, the shear force of the slurry far from the surface of the screw will gradually decrease, so the velocity of the slurry closer to the surface of the screw will be greater. In addition, it can be seen in Figure 7 that the velocity distribution of the concrete slurry in the conveying section of the screw has almost no obvious change, which indicates that the screw can keep the speed stable during the extrusion of the slurry and play a certain role in speed regulation.

**Figure 12:**
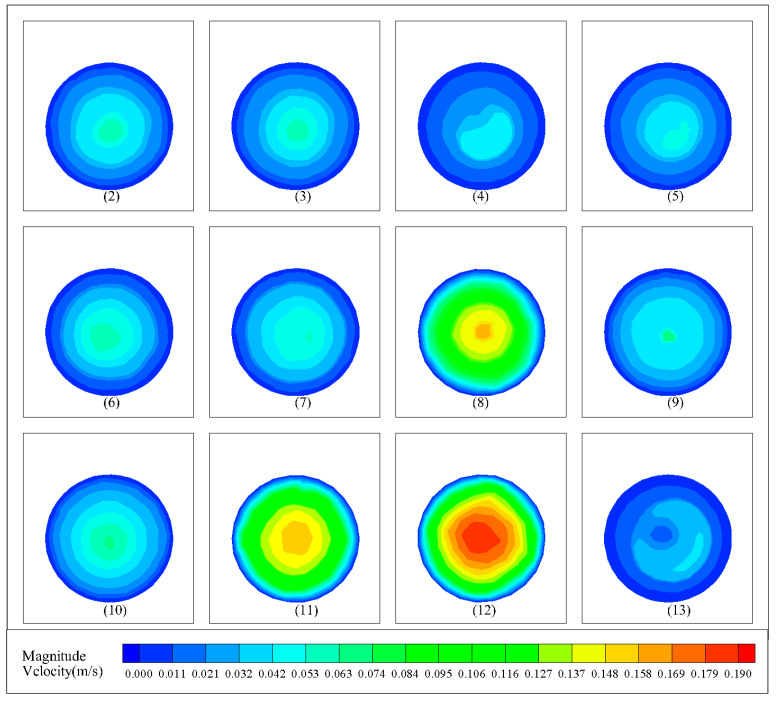
The remaining 12 groups of simulated outlet cross-section velocity contours.

Figure 12 presents the velocity cloud diagram of the exit section of the other 12 groups of simulation results. It can be seen that there are some differences in the simulation results of different parameter combinations. However, in general, the exit velocity gradually decreases from the center to the outside, with the maximum velocity appearing at the exit center and the minimum velocity appearing at the exit wall. This is because the friction force of the inner wall of the nozzle when the concrete is extruded from the nozzle is encountered. As a result, the concrete speed near the inner wall of the nozzle is smaller, and the speed at the center is larger, resulting in uneven speed at the exit.

In order to make the velocity at the exit section as uniform as possible, the standard deviation of the velocity at each group of exit sections is extracted, which is used as the optimization objective. The smaller the standard deviation is, the more uniform the velocity is. This can ensure the more uniform the velocity of the exit section when the concrete is extruded from the nozzle and ensure the print quality. The orthogonal test results of each group are shown in Table 6.

#### 3.3.5. Optimization of Printing Nozzle Structure Parameters

It can be seen in Figure 7 that the extrusion system has three important parameters, including inlet diameter *D*, nozzle inclination angle *θ*, and outlet length *L*, which determine the extrusion characteristics of the nozzle. This paper adopts response surface methodology to optimize the nozzle structure.

The sequential quadratic programming (SQP) algorithm is an effective algorithm for solving constrained optimization problems of small- and medium-sized programs. Its basic idea is to transform nonlinear optimization problems into a series of quadratic programming subproblems and gradually approach the optimal solution of the original equation by solving these subproblems, as expressed below [19]:

(3) $$\min\nabla f{\left(x_{k}\right)}^{T}d+\frac{1}{2}d^{T}H_{k}d$$

(4) $$s.t.\;c_{i}\left(x_{k}\right)+\nabla c_{i}{\left(x_{k}\right)}^{T}d=0,i\in E$$

(5) $$c_{i}\left(x_{k}\right)+\nabla c_{i}{\left(x_{k}\right)}^{T}d\geq 0,i\in I$$

**Figure 13:**
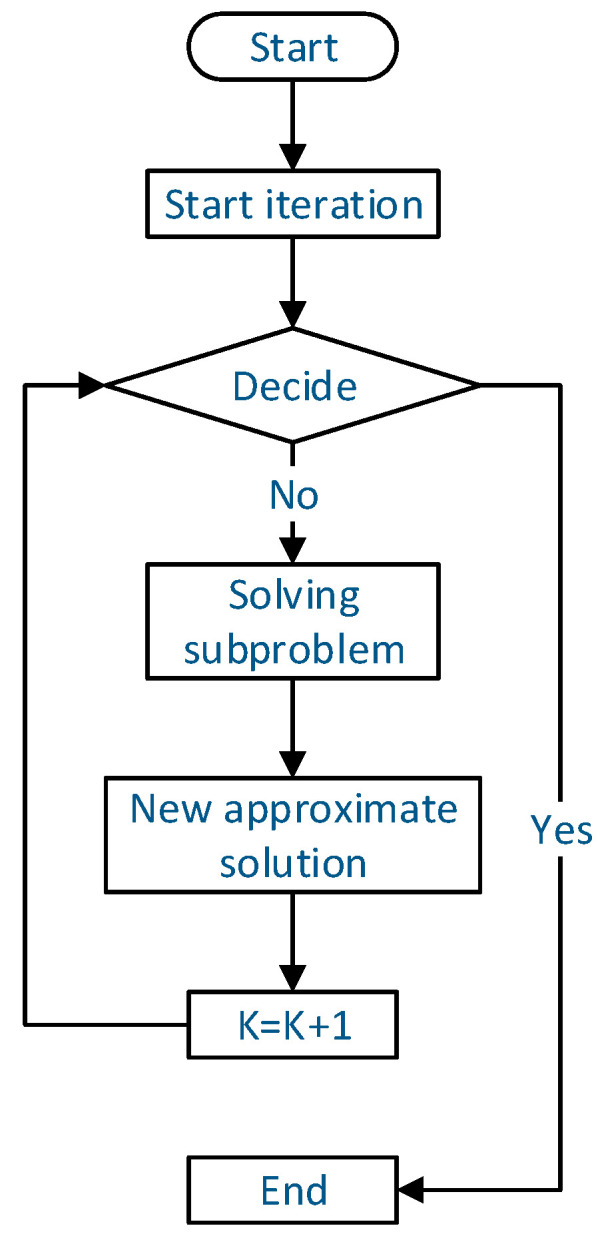
The flow chart of SQP method to solve the problem.

Its solution process is shown in Figure 13. Here, $x_{k}$ is the iteration point of the current problem, and the linear programming subproblem is obtained by linearization near the current iteration point. The search direction $d_{k}$ of the current iteration point is obtained by solving the subproblem, and the step $\;a_{k}\;$ is determined. The next iteration point $x_{k+1}=x_{k}+a_{k}d_{k}\;$ is updated according to the search direction, and the values of the new objective function and constraint function are calculated. Then, it is determined whether the stop criterion is met. If it is met, the iteration will be stopped; otherwise, return to the first step to continue the iteration [20].

Input the extracted results of the speed standard deviations for each group into the Design-Expert 11 software and then proceed to analyze the data. Various models were utilized to fit the data, and the model with the smallest fitting error was selected. The fitting results for each model are presented in Table 7 below.

It can be seen in Table 4 that the 2FI model has high fitting and prediction accuracy at the same time. Therefore, the 2FI model is selected to fit the data, and the influence of three factors, namely, inlet diameter *D*, nozzle inclination *θ* and outlet length *L*, on the standard deviation Std *V* of the exit section velocity of the response value is analyzed. The equation after fitting the 2FI model is as follows:

(6) $$Std\;V\times 10^{3}=-74.6+1.9\times A+1.7\times B+2.6\times C-0.051\times AB-0.089\times AC+0.1\times B$$

where *ABC* represents the inlet diameter *D*, the nozzle inclination angle *θ*, and the outlet length *L*, respectively. The results of ANOVA fitting are shown in Table 8.

From Equation (6), it can be seen that the coefficient of *C* is the largest, and the coefficients of A and B have a small difference, indicating that the outlet length L has the greatest influence on the standard deviation of the outlet section velocity, followed by the inlet diameter D. The nozzle inclination angle *θ* has the least influence.

**Figure 14:**
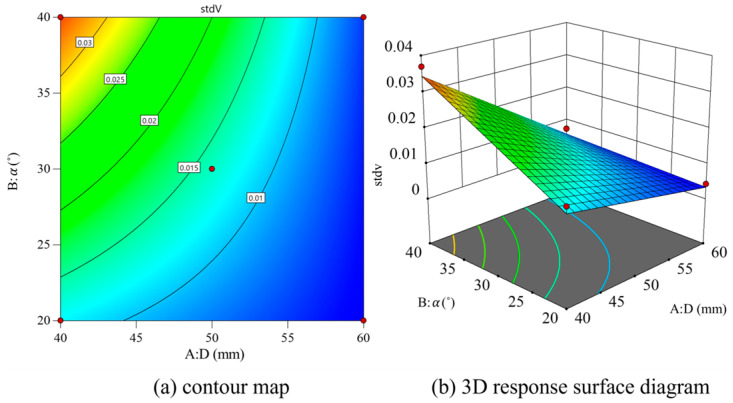
Contour map and 3D response surface map of *D* and *θ*.

With *D* and *θ* as variables, and the values of *L* fixed, contour and 3D surface maps are drawn as shown in Figure 14. As the inlet diameter increases, the standard deviation of exit section velocity Std *V* gradually decreases. In addition, Std *V* increases gradually with the increase in nozzle inclination. As can be seen in the 3D surface diagram, Std *V* can be significantly reduced when the inlet diameter is small. When the inlet diameter exceeds 50 mm, Std *V* decreases more and more slowly. On the contrary, Std *V* can be significantly reduced under a larger nozzle inclination.

**Figure 15:**
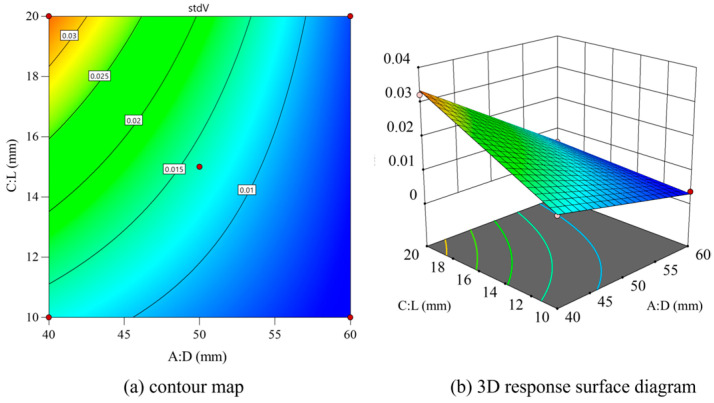
Contour map and 3D response surface map of *D* and *L*.

With *D* and *L* as variables, and the *θ* values fixed, contour and 3D surface maps were drawn as shown in Figure 15. Std *V* gradually increased with the increase in inlet diameter, and Std *V* gradually increased with the increase in *L*. It can be seen in the 3D response surface diagram that the outlet length can reduce Std *V* in small cases; however, Std *V* increases more obviously when the nozzle length increases.

**Figure 16:**
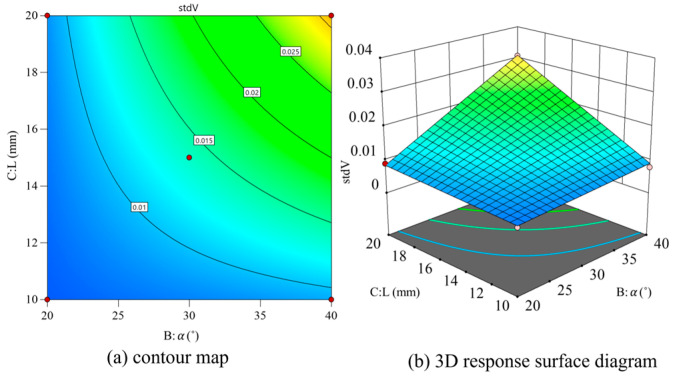
Contour map and 3D response surface map of *θ* and *L*.

With *θ* and *L* as variables and fixed *D* values, contour and 3D surface maps are drawn as shown in Figure 16. Std *V* gradually increases with the increase in the nozzle inclination angle and outlet length. As can be seen in the 3D response surface diagram, Std *V* can be significantly increased when the nozzle inclination and outlet length are both large values. When they are gradually reduced, Std *V* also gradually decreases, and the reduction amplitude is gradually smaller.

The optimization of printing nozzle parameters is a multi-variable, constrained, and nonlinear minimization problem. In order to make the extruded concrete more uniform, the optimization goal is to minimize the velocity standard deviation of the exit section. Then the objective function is expressed as follows:

(7) $$\min f\left(D,\theta ,L\right)=Std\;V$$

The value range of inlet diameter *D*, nozzle inclination angle *θ*, and outlet length *L* is expressed as follows:

40≤D≤60;20°≤θ≤40°;10≤L≤20;

In addition, the value of Std *V* should be positive, so the value range of *std V* is expressed as follows:

Std V≥0

Input the fitted equation for the velocity standard deviation of the exit section, along with the value range of each parameter, into the Optimization Toolbox in MATLAB R2022b to solve this problem. Set the initial point as (40, 30, 20), and obtain the optimal solution as follows: *D* = 55 mm, *θ* = 20°, *L* = 34 mm. Here, the objective function value Std *V* is 1.9 × 10^−3^.

**Figure 17:**
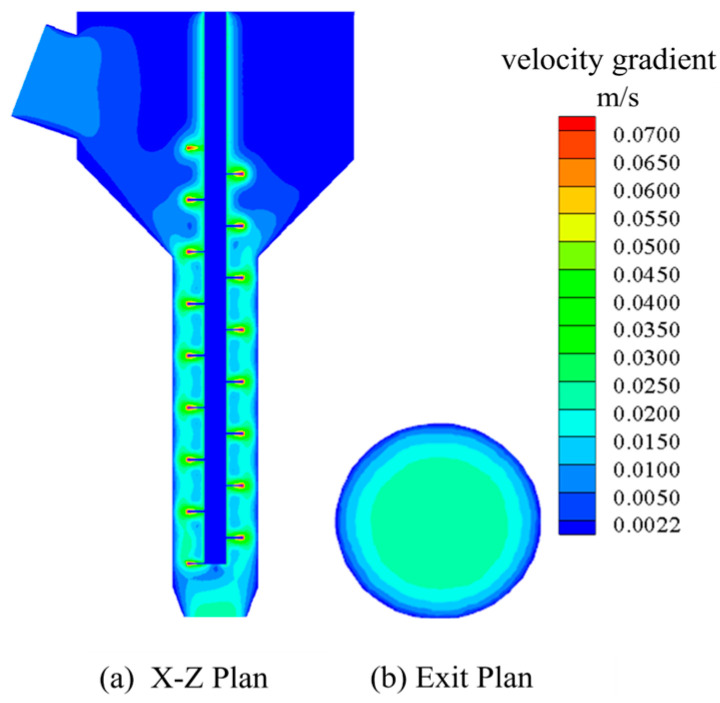
Calculation results of optimal structural parameter model.

The extrusion mechanism model with optimal structural parameters was established for simulation, and the parameter settings were the same as above. The calculation results of optimal structural parameters are shown in Figure 17 below.

Figure 17a shows the velocity distribution of the X-Z plane section, and Figure 17b shows the velocity distribution of the exit section. The velocity standard deviation of the exit section was extracted and compared with the group with the smallest velocity standard deviation in Table 3. The comparison results are shown in Table 9.

It can be seen in Table 6 that the velocity variance of the exit section of the optimized scheme is smaller than the result of the optimal scheme in the test in Table 9. Specifically, it is reduced by 34.8%, which indicates that the optimization of nozzle structure parameters using this method is effective.

#### 3.3.6. Extrusion Test

**Figure 18:**
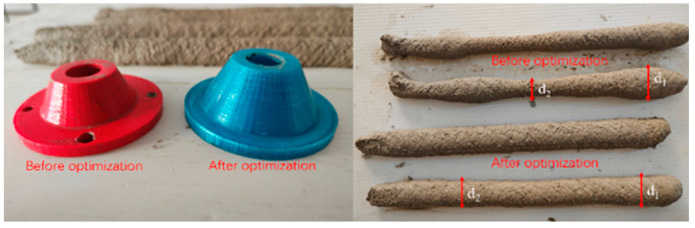
Comparison results before and after optimization.

A self-made concrete 3D printer was used to install the circular nozzle printing nozzle before and after optimization on the self-made frame concrete 3D printer. Before the test, the silo and nozzle were moistened with water. After the water was completely drained, the evenly mixed slurry was loaded into the printer, and the spiral shaft was started. The length of the printed specimen was designed to be 40 cm. The printed specimen after completion is shown in Figure 18, the largest and smallest transverse widths *d*_1_ and *d*_2_ were measured, respectively. The difference *d*_1_–*d*_2_ was used to represent the uniformity of extrusion. For the data pairs before and after optimization, as shown in Table 10, after measurement, the difference *d*_0_ after optimization is smaller than the difference before optimization, indicating that this method is effective.

## 4. Influence of Printing Process Parameters on Forming Quality and Performance

**Figure 19:**
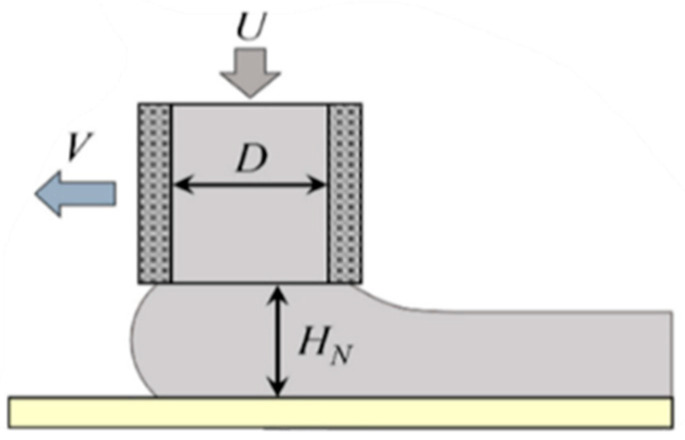
Concrete 3D printing schematic diagram.

In the concrete 3D printing process, the extrusion speed *U* (affected by screw speed), printing speed *V*, nozzle height *H_N_*, nozzle diameter *D*, and other parameters have significant impacts on the printing effect. When the screw speed increases, the extrusion speed *U* increases, and the extruded concrete material increases. If the printing speed is too fast, the material may break and affect the continuity. If the nozzle height *H_N_* is too small, the printing strip width will be increased, and the printing accuracy will be affected. Therefore, in order to obtain better 3D printing forming quality, it is necessary to reasonably select these parameter combinations. Using an orthogonal experimental design, the optimal combination of process parameters can be found effectively. Figure 19 presents a diagram of the concrete 3D printing process.

### 4.1. Material Performance Index

**Figure 20:**
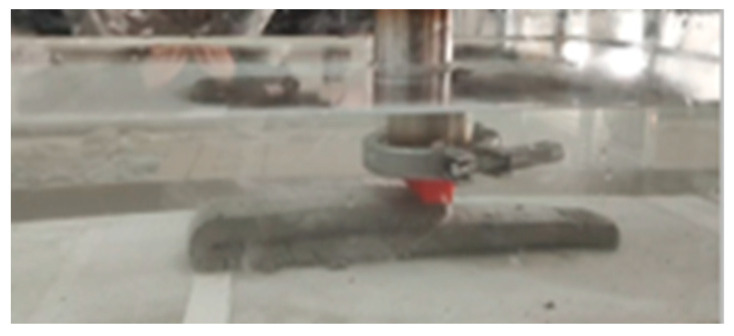
Concrete 3D printing underwater.

The underwater 3D printing process for the R-0 group, as illustrated in Figure 20, exhibits a smooth surface and excellent buildability throughout the printing process, without any noticeable material dispersion. The anti-dispersion properties of the R-0 group material are presented in Table 11, and the experimental results for buildability are shown in Table 12. The compressive and flexural strengths are 24 MPa and 8.5 MPa, respectively. The moderate compressive and flexural strengths after 7 days indicate that this mix proportion is suitable for underwater 3D printing.

### 4.2. Process Parameter Test Scheme

On the basis of R-0, an orthogonal experiment is used to design the scheme. Printing speed, nozzle height, and screw speed were considered as three factors, and each factor was set at three different levels, as shown in Table 13.

Using the orthogonal test table designed for three factors, each with three levels, various combinations of factor levels were arranged as presented in Table 14. Experiments were then conducted according to the specified groups outlined in the experimental table, with the results meticulously recorded.

### 4.3. Test Results and Analysis

#### 4.3.1. Forming Quality Analysis

**Figure 21:**
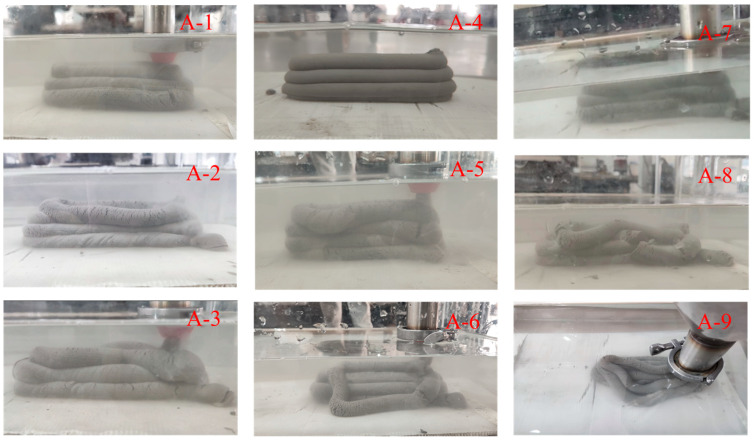
A1–A9 underwater printing test results.

The process of underwater printing tests for each group was recorded, and the test results of groups 1 to 9 are shown in Figure 21. As can be seen in Figure 21, the third component has poor quality, while the first component has good quality. This is because when the printing speed is 12 mm/s and the height of the nozzle head is high, the concrete in the water experiences uneven forces during extrusion due to buoyancy and water pressure. These uneven forces cause the extruded strip concrete to shift, resulting in collapse and deformation. The higher the height of the nozzle is, the easier it is to make the strip of concrete offset in the falling process. At this time if the screw speed is small, the concrete is not enough to supply extrusion, which will aggravate the occurrence of offset and affect the forming quality.

The fourth component has the best quality. The surface is smooth without cracks and does not collapse, while the strip concrete from the sixth group has collapsed and shifted. This is because the three process parameters of the fourth group are more matched, so the concrete can be uniformly and stably extruded. In addition, the appropriate printing speed ensures that the concrete extruded from the nozzle is properly discharged in time, preventing the concrete from gathering at the exit. Thus, the concrete shows a uniform strip. The nozzle height of the sixth group is too high, and the screw speed is too small. This results in an insufficient supply of concrete extrusion. A phenomenon where the nozzle drags the materials will occur, causing deviation in the concrete strip and resulting in the final collapse. The printing effect of group 5 is improved compared with that of group 6. However, there is a slight deviation when the concrete bars are stacked, and the bars are thickened locally at the turning and lifting places due to excessive screw speed. The printing effect of group 7 is better, and the bending deformation of the concrete strip occurs during the printing process in the other two groups. The reason is that the nozzle height of groups 8 and 9 is high, and the screw speed is small. Thus, the concrete supply is not provided in a timely fashion, and the pulling deviation occurs during extrusion with the final collapse.

In summary, when the moving speed and screw speed are too fast or too slow, it will affect the forming quality of the specimen, which may cause the specimen to break or collapse in serious cases. According to the observation of the above nine groups of experiments, the printing effect is better in groups 1, 4, and 7, of which group 4 has the best printing effect.

#### 4.3.2. Mechanical Properties Analysis

**Figure 22:**
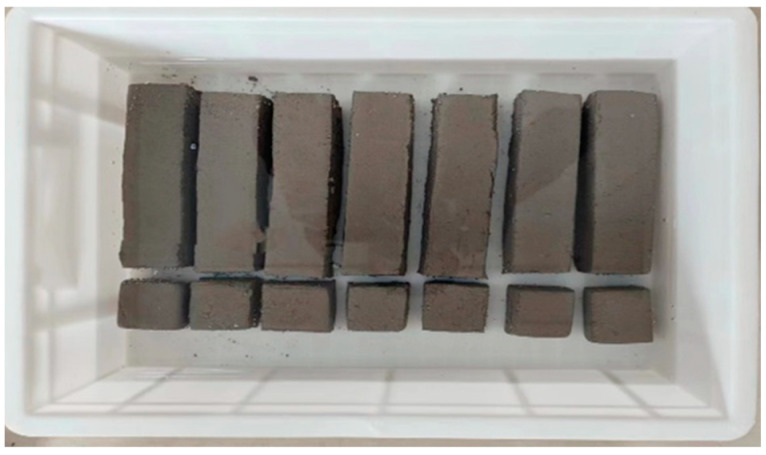
The compressive and flexural strength test specimens.

**Figure 23:**
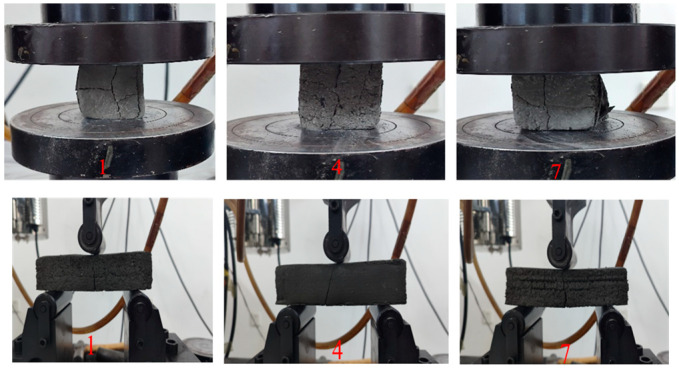
Compression (**top**) and bending (**bottom**) failure modes of the specimen.

The above three groups of specimens were cured in water for 7 days. The compressive and flexural specimens are shown in Figure 22. Their compressive and flexural strengths were measured. The compression and bending failure patterns of each group are shown in Figure 23.

It can be seen in Figure 23 that the failure patterns of the three groups of specimens are different. In the compression process of the first group of specimens, two cracks running through the upper and lower direction appear in the vertical direction, and two short cracks appear in the horizontal direction. This is because the printing process parameters of this group are as follows: the printing speed is 12 mm, the printing height is 10 mm, and the screw speed is 40 r/min. At this time, the screw speed is small and is not enough to supply the extrusion of the slurry. The pressure on the surface of the material at the exit is small, and the interlayer bonding ability is weakened. Thus, cracks will occur in the horizontal direction of the layer. Although the screw speed of group 7 is increased to 60 r/min, its printing speed is also increased to 20 mm/s. At this time, the printing speed is increased greatly, which will also lead to insufficient material supply. However, there is a slight improvement compared with group 4. Therefore, the horizontal and vertical cracks in the specimen are reduced. The process parameters of group 4 are a printing speed of 16 mm, printing height of 10 mm, and screw speed of 50 r/min. At this time, the three parameters match reasonably. The material can be timely and evenly extruded, and the interlayer pressure provided during the extrusion process is stable. Thus, the interlayer bonding ability is enhanced. Therefore, only vertical cracks exist in the printed specimen, and the cracks are minimal. In addition, the crack trend of the three groups of specimens in the bending process is basically the same, and all of them extend from the middle bottom to the upper slope until the specimen breaks.

**Figure 24:**
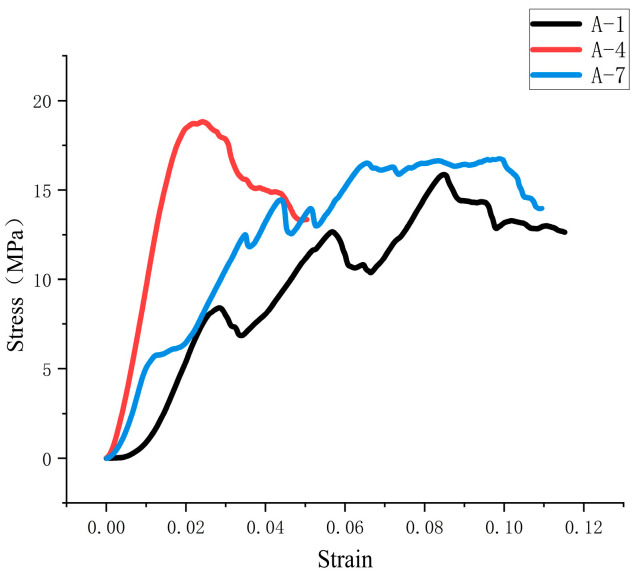
Stress–strain curve of group A specimen during compression.

**Figure 25:**
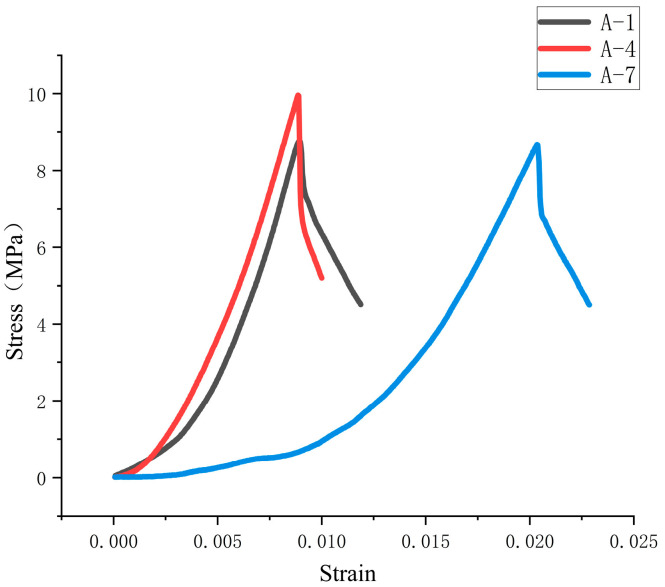
Stress–strain curve of group A specimen during bending.

**Figure 26:**
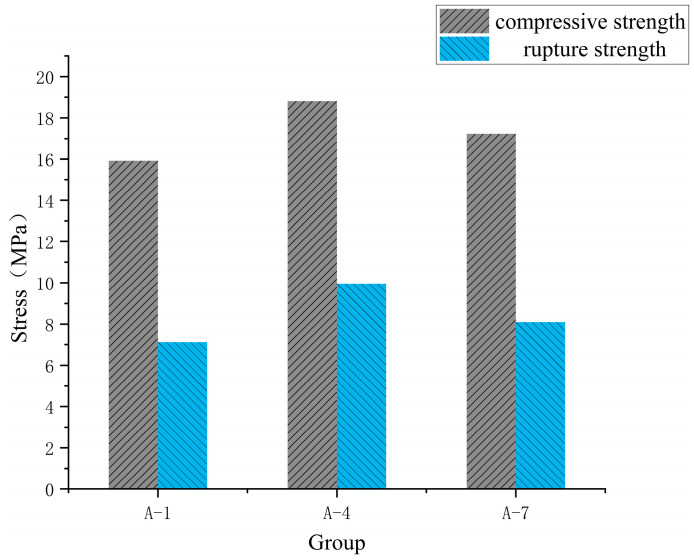
Compressive and flexural strength of the specimen.

As shown in Figure 24, the stress curves of groups 1 and 7 oscillate during the rising process because the screw speed does not match the amount of slurry extruded. This results in a small pressure on the surface of the material at the outlet and a weakening of the interlayer bonding ability. As shown in Figure 25, due to the large increase in the printing speed of group A-7, the bonding strength between layers is low, and the internal voids are increased, resulting in abnormal delay of the stress peak. The measurement results of compressive and flexural strength of specimens in each group are shown in Figure 26. It can be seen in the figure that different printing process parameters have an impact on compressive and flexural strength. The compressive strength and flexural strength of group 4 are the largest, which are 18.81 MPa and 9.96 MPa, respectively. The combination of printing process parameters is as follows: printing speed is 16 mm/s, nozzle height is 10 mm, and screw speed is 50 r/min. The use of a nozzle with a circular outlet will result in a lower compressive flexural strength than that printed using a rectangular nozzle [21].

In summary, the nozzle height is very important for printing. A nozzle height that is too high will lead to weak interlayer bonding and weaken the mechanical properties of the specimen. A nozzle height that is too low makes it difficult to form and easily leads to experimental failure. It is worth noting that when the process parameters change, the bending strength changes more significantly. Thus, the process parameters have a greater impact on the bending strength.

## 5. Discussion

By comparing research on 3D printing of concrete in air, it can be observed that process parameters such as printing speed, layer thickness, and nozzle size have significant impacts on the molding quality and mechanical properties of printed parts [22,23,24,25]. These parameter optimization strategies in air have been extensively studied and validated. However, when these parameters are applied in an underwater environment, optimizing them becomes more complex and challenging due to factors such as water buoyancy, resistance, and the unique behavior of underwater concrete materials. Table 15 briefly summarizes the current settings of nozzle parameters and printing process parameters in air.

When conducting underwater 3D printing of concrete, in order to effectively address the vortex effects generated by the movement of the nozzle, the printing speed is significantly reduced compared to that in air. Compared to the three printing speeds of 70 mm/s, 90 mm/s, and 110 mm/s proposed in Panda’s research [15], the printing speed presented in this paper is only 16 mm/s, representing a significant difference. However, the process of underwater 3D printing of concrete involves not just the single factor of printing speed, but rather a complex interplay among printing speed, nozzle height, and screw rotational speed. Additionally, the underwater construction environment and the anti-dispersion performance of concrete materials underwater must also be comprehensively considered. In concrete 3D printing, the setting of nozzle height is closely related to the nozzle outlet diameter or cross-sectional area. For example, Wolfs defaults the printing height to be equal to the width of the nozzle cross-section. Wen Z determined that the parameter for a single-layer height is generally about half the diameter of the nozzle. In this study, the nozzle diameter used for underwater printing experiments was 20 mm, and the final nozzle height was 10 mm, which aligns with Wen Z’s suggestion that the single-layer height should generally be half of the nozzle diameter. Due to the complex and variable underwater environment, coupled with the significant difference in the curing time of concrete materials underwater compared to that in air [8], setting the nozzle height with greater precision is necessary to avoid adverse effects on the final print quality from interlayer bond strength during underwater printing. This precise setting is crucial for ensuring the quality of underwater 3D-printed concrete. The screw rotational speed, which is closely related to the feed rate of 3D-printed concrete, has an optimal matching relationship with the movement speed of the nozzle. When the movement speed and screw rotational speed reach the optimal ratio, the molding accuracy of the printed structure will be the highest. Therefore, based on comprehensive consideration of movement speed and nozzle height, this study obtained the optimal screw rotational speed parameter through process monitoring and photographic analysis of underwater 3D orthogonal experiments. The determination of this parameter is of great significance for improving the quality and efficiency of underwater 3D-printed concrete.

Although these trends are similar to those observed in 3D printing of concrete in air, there are significant differences in specific numerical ranges and optimal parameters. This further highlights the uniqueness and complexity of optimizing process parameters for underwater 3D printing of concrete.

In summary, although there are relatively few relevant literature on underwater 3D printing of concrete, by comparing and analyzing related research on 3D printing of concrete in air, we can initially understand the impact of process parameters on the molding quality and mechanical properties of underwater 3D-printed concrete. These research results provide useful insights for the future development of this field and valuable references and guidance for subsequent researchers.

## 6. Conclusions

In this paper, using numerical simulation and experiments, the influence of nozzle structure parameters on the exit speed is studied, and its structure parameters are optimized to improve the printing accuracy and surface quality. At the same time, the influence of screw speed, printing speed, and nozzle height process parameters on the 3D printing quality of underwater concrete is discussed. The following key conclusions are drawn based on these experiments:

1. The outlet length *L* has the greatest influence on the exit section speed. The inlet diameter *D* has the second greatest influence, and the nozzle inclination angle *θ* has the least influence. The velocity variance of the optimized scheme is smaller than that of the optimal scheme in the experiment, and it is reduced by 34.8%. Finally, the structural parameters that minimize the standard deviation of the velocity of the outlet section are obtained: *D* is 55 mm, *θ* is 20°, and *L* is 34 mm.
2. During the printing process, if the nozzle height is too high, it will cause the material to be not tightly combined in the middle layer of the printing process, thus weakening the mechanical properties of the material and even causing collapse in severe cases. On the contrary, if the nozzle height is too low, the material will be hindered during the extrusion process, resulting in poor printing and overflow on both sides of the material. This will directly affect the effectiveness of the constructability evaluation index of the material and then affect the forming quality of the underwater 3D-printed concrete.
3. In the printing process, a screw speed that is too fast speed will lead to excessive extrusion of the material, which will cause deviation of the underwater 3D-printed concrete during the stacking process. This will lead to the collapse of the material. On the contrary, if the screw speed is too slow, it will lead to a shortage of the extrusion supply of the printing materials. The nozzle will drag the material, which will prevent the material from forming or the forming quality will be poor. The material fracture will seriously affect the final forming quality.
4. In the printing process, if the moving speed is too fast, the material will easily form cracks. This will cause the internal cracks of the formed material to increase, thus weakening its mechanical properties and even causing the material to fracture in serious cases. On the contrary, if the movement speed is too slow, the material may collapse due to instability during the accumulation process. Thus, ultimately, the formation of a complete structure is not possible.
5. To sum up, the printing effects of groups 1, 4, and 7 were determined to be better using underwater printing experimental verification. The compressive and flexural strength of the three groups of specimens is measured. The combination of the process parameters of group 4 provide the best comprehensive effect. Specifically, the printing speed is 16 mm/s, the height of the nozzle is 10 mm, and the screw speed is 50 r/min.

## Figures and Tables

**Table 1:** Basic mix proportion of underwater 3DPC.

Group	Cement	Mineral Powder	Fly Ash	Quartz Sand	Alkaline Silica Sol	Water	HPMC	WR	Li_2_CO_3_
R-0	200	100	100	100	9	115	0.25%	0.38%	0.10%

**Table 2:** Main indexes of cement.

Breed	Specific Area m^2^/kg	Rupture Strength/MPa		Compressive Strength/MPa		Setting Time/min	
		1d	3d	1d	3d	Initial	Final
R.SAC 42.5	410	6.1	6.5	37.2	45.1	10	15

**Table 3:** Print nozzle parameter range values.

Influence Factor	Level		
Inlet diameter *D* (m)	0.04	0.05	0.06
Nozzle angle *θ* (°)	25	27.5	30
Outlet length *L* (m)	0.02	0.03	0.04

**Table 4:** Test design results.

Group	*D*/m	*θ*/°	*L*/m
1	0.05	30	0.03
2	0.04	30	0.03
3	0.06	25	0.03
4	0.04	27.5	0.04
5	0.06	27.5	0.04
6	0.04	25	0.03
7	0.05	27.5	0.03
8	0.05	25	0.04
9	0.05	30	0.04
10	0.06	30	0.03
11	0.04	27.5	0.02
12	0.06	27.5	0.02
13	0.05	25	0.03

**Table 5:** Rheological equations.

Model	Equation	R^2^
Bingham	$\tau =\tau_{0}+\mu \gamma$	0.828
Power Law	$\tau =K\gamma^{n}$	0.951
Modified Bingham	$\tau =\tau_{0}+\mu \dot{\gamma }+c\gamma^{2}$	0.978
H–B	$\tau =\tau_{0}+K\cdot \gamma^{n}$	0.997

**Table 6:** Results of the orthogonal test.

Group	*D*/mm	*θ*/°	*L*/mm	Max Speed *V*/(m/s)	Std *V*/(10^−3^)
1	60	30	20	0.0742107	4.27177
2	50	30	20	0.0742346	7.84823
3	50	25	30	0.0742344	9.5398
4	50	27.5	20	0.0742356	6.14242
5	60	27.5	20	0.074489	6.92645
6	50	25	40	0.074491	8.95327
7	60	27.5	30	0.0744899	7.25807
8	40	25	40	0.1506329	32.15794
9	40	30	40	0.0881407	13.58164
10	40	30	10	0.0879261	12.37306
11	50	27.5	20	0.1548642	30.69799
12	40	27.5	20	0.189449	36.99066
13	60	25	30	0.0744895	3.652843

**Table 7:** Fitting results of each model.

Model	*p* Value	Fitted R^2^ Values	Predicted R^2^ Value
Linear	0.0017	0.7337	0.5707
2FI	0.0029	0.9552	0.9243
Quadratic equation	0.0779	0.9882	/

**Table 8:** The variance analysis results of the standard deviation of the exit cross-section velocity.

Source	Sum of Squares/10^−6^	Degree of Freedom	Mean Square/10^−6^	F Value	*p* Value
Regression model	1600	6	300	43.66	0.0001
Factor *A-D*	700	1	700	115.69	<0.0001
Factor *B-θ*	300	1	300	51.83	0.0004
Factor *C-L*	300	1	300	46.9	0.0005
Factor *AB*	100	1	100	17.41	0.0059
Factor *AC*	100	1	100	13.33	0.0107
Factor *BC*	100	1	100	16.78	0.0064
Residual	0	6	5.99	/	/
Aggregate	1600	12	/	/	/

**Table 9:** Scheme comparisons.

Comparison Test	*D*/mm	*θ*/°	*L*/mm	Std *V*/10^−3^
Optimal scheme in the test	50	25	30	3.652843
Optimized scheme	55	20	34	2.381934

**Table 10:** Data comparison before and after optimization.

*D*/mm	After Optimization/mm	Before Optimization/mm
*d*_1_	2.35	2.48
*d*_2_	2.21	1.91
*d*_0_	0.14	0.57

**Table 11:** Dispersion resistance.

Group	Mass Loss Rate/%	pH Valve			
		5 min	10 min	20 min	30 min
R-0	1.17	8.21	8.35	8.91	9.26

**Table 12:** Results of the constructability test.

Group	High/mm	Upper Base Width/mm	Lower Base Width/mm	tan *a*	Cross-Sectional Area Ratio
R-0	75	60	45	10	1.23

**Table 13:** The value of process parameters.

Influence Factor	Level		
Printing speed *V* (mm/s)	12	16	20
Nozzle height *H* (mm)	10	12	14
Screw speed *ω* (r/min)	40	50	60

**Table 14:** Orthogonal experiment table.

Group	Factor		
	Printing Speed *V* (mm/s)	Nozzle Height *H* (mm)	Screw Speed *ω* (r/min)
A-1	12	10	60
A-2	12	12	40
A-3	12	14	50
A-4	16	10	40
A-5	16	12	50
A-6	16	14	60
A-7	20	10	50
A-8	20	12	60
A-9	20	14	40

**Table 15:** Setting of process parameters based on the existing literature.

Researchers	Key Conclusions Presented
Tay [13]	A parameter SR is introduced to represent the influence of the printing parameters on the cross-section shape. It is found that when the SR is equal to 1, the printed mortar strip is better.
Wolfs [22,23]	The default print height should be equal to the nozzle section width.
Ma [24]	The study of Ma et al. shows that the width of the extruded strip decreases with the increase in the yield stress of the cement-based material at the same printing speed.
Kruge [25]	It is found that there is an optimal combination of the printing speed and the height between the printing layers that can ensure the precision of the printing structure and improve the printing efficiency.
Zhou W [26]	The height of the single layer is generally taken to be about half of the diameter of the nozzle.

## Data Availability

The original contributions presented in this study are included in the article. Further inquiries can be directed to the corresponding author.
